# Separation of Human Monocytes from a Leukocyte-Rich Plasma

**DOI:** 10.1100/tsw.2002.843

**Published:** 2002-06-15

**Authors:** John Graham

**Affiliations:** School of Biomolecular Sciences, Liverpool John Moores University, UK

**Keywords:** human monocytes, OptiPrep™, iodixanol, leukocyte-rich plasma, density barrier, CD14, CD4

## Abstract

Human peripheral blood monocytes are isolated by flotation from a dense leukocyte-rich plasma (LRP) through two lower-density barriers prepared from OptiPrep™. The separation from lymphocytes depends on the more rapid rate of flotation of the monocytes because of their slightly lower density and larger size. The method works optimally only with fresh (within 2 h of drawing) EDTA-anticoagulated blood.

**METHOD TYPE:** extraction, isolation, purification and separation

**SUB METHOD TYPE:** centrifugation

## INTRODUCTION

The monocytes in human peripheral blood, account for, on average, about 8% of the leucocyte population. They tend to be larger (15–20 μm) than lymphocytes (6–20 μm) and they also have a slightly lower density (Fig. 1). These properties allow some scope for their separation by centrifugation[1]. If a buffered OptiPrep™ Stock ($\rho = 1.317$ g/ml) is added to a leucocyte-rich plasma (LRP) to raise its density to approx. 1.1 g/ml, the leukocytes will float to the top of the plasma (Fig. 2) when this suspension is centrifuged.

In this way the mononuclear cells rapidly form a narrow band at the interface between the sample and a 1.084-g/ml solution layered on top. The monocytes (because of their size and

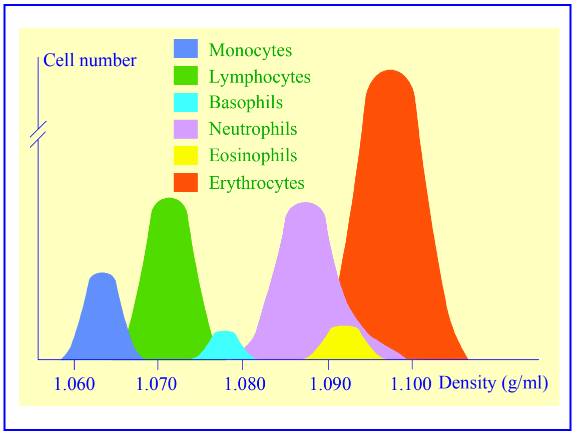

**FIGURE 1.** Density of human blood cells.

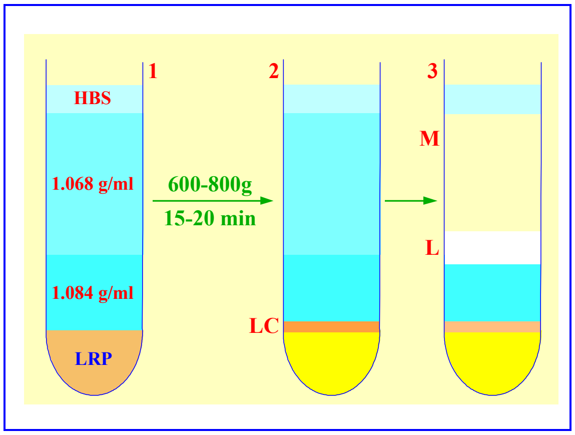

**FIGURE 2.** Isolation of human monocytes: LRP = leucocyte-rich plasma; LC = leucocytes; HBS = Hepes-buffered saline-BSA. LC form a sharp band during centrifugation (1-2) from which monocytes (M) and lymphocytes (L) separate by flotation.

density) migrate upwards through this layer and through a second low-density barrier ($\rho$ = 1.069g/ml). The smaller and denser lymphocytes tend to float more slowly, and in this way a separation between the two types of cells is effected. The cells remaining at the top of the load zone are granulocyte-enriched and the protocol has been used for the simultaneous isolation of monocytes, lymphocytes, and granulocytes[2]. For other published papers in which OptiPrep™ was used to isolate monocytes, see Refs. [3,4,5,6,7].

## MATERIALS AND EQUIPMENT

OptiPrep™ (shake gently before use)
Diluent: 100 mM EDTA, 1M Hepes-NaOH, pH 7.4
Hepes-Buffered Saline/Bovine Serum Albumin (HBSA): 0.85% (w/v) NaCl, 1 mM EDTA, 10 mM Hepes-NaOH, pH 7.4, containing 0.5% (w/v) bovine serum albumin (make up fresh)
Plastic screw-capped conical centrifuge tubes (12–15 ml, see Note 1)
Plastic Pasteur pipette for overlayering
Low-speed (temperature-controlled) centrifuge with swinging-bucket rotor

## METHOD

1. Shake the OptiPrep™ gently before use.
2. Prepare a buffered OptiPrep™ Stock by adding 0.1 vol of Diluent to 10 ml of OptiPrep™.
3. Prepare two solutions of 1.068 g/ml and 1.084 g/ml by mixing solution the OptiPrep™ Stock and HBSA as follows: 2.02 ml + 7.98 ml and 2.51 ml + 7.49 ml, respectively (see Note 2).
4. Centrifuge freshly drawn, whole blood (anticoagulant 1 mM EDTA) at $200g_{av}$ in a swinging-bucket rotor, at about 20°C, for 10–15 min (see Note 3).
5. Harvest the buffy coat in the plasma supernatant (LRP). Some erythrocytes will also be collected but try to keep them to a minimum. Over 80% of the leucocytes are recovered in this manner.
6. Mix the LRP with OptiPrep™ Stock (2 vol + 0.8 vol, respectively) and overlayer approx. 2.0 ml with 2.0 ml of the 1.084 g/ml solution and 4.0 ml of the 1.068 g/ml solution and then layer a small volume of HBSA (approx. 0.5 ml) on top (see Notes 4 and 5).
7. Centrifuge at $600–800g_{av}$ in a swinging-bucket rotor for 15–20 min at 20°C. Do not use the brake during deceleration (see Note 6).
8. Collect the monocytes that float into the 1.069 g/ml layer (Fig. 2 and Note 7).

## NOTES

1. The type of centrifuge tube that is used is important as monocytes tend to adhere to some surfaces and if this occurs the cells tend to become activated. Because most purifications require the monocytes to be isolated under sterile conditions in sealed tubes, plastic tubes are obviously the tubes of choice. Some plastics are more prone to cause monocyte adhesion than others; Graziani-Bowering et al.[1] used polypropylene tubes. Because of the huge range of plastic centrifuge tubes now available commercially, it is impossible to give any firm recommendations and it is strongly advised that the tubes be checked for this problem before use.
2. As this method separates the monocytes and lymphocytes on the basis of density <u>and</u> size, small differences in run conditions from laboratory to laboratory may influence its success. Improved recoveries of monocytes may be obtained by adjusting the density of the 1.084-g/ml layer within the range 1.079–1.089 g/ml (see Ref. [8] for information on solution preparation). There is evidence that it may be preferable to prepare the two density gradient solutions by diluting the OptiPrep™ Stock with a culture medium (RPMI or DMEM) containing 10% serum. The small increase in the density of two solutions (an increase of approx. 0.002 g/ml) may also be beneficial.
3. The method only works satisfactorily on fresh blood (used within 2 h of drawing) from healthy individuals, using EDTA as an anticoagulant.
4. Larger volumes should be processed in larger tubes while maintaining a similar geometry of sample and low-density barriers. In 50-ml tubes for example, 14 ml of sample and 7.5 and 20 ml of the 1.084 and 1.069 g/ml density solutions have been successfully used[1].
5. The uppermost layer of medium is not required for the separation process, but it avoids the banding of cells at a liquid/air interface and also prevents the cells from adhering to the walls of the tube at the meniscus.
6. Increasing the time of centrifugation will increase yields but decrease purity. If lymphocyte contamination is unacceptable, try reducing the centrifugation time or harvest less of the 1.069-g/ml layer or make the middle density barrier 1.079 g/ml rather than 1.084 g/ml.

7. Because of small variations in tube sizes and centrifugation conditions the precise position of the monocyte band may vary. The monocyte band isolated by this technique has been analyzed by flow cytometry and contains at least 90–95% monocytes with only 3–5% contamination from T-cells. Results from a typical experiment are given in the table below.

| Surface Marker | % events |
|---|---|
| CD3+ | 3.4 |
| CD14+/CD4- | 1.6 |
| CD14-/CD4+ | 6.9 |
| CD14+/CD4+ | 84.1 |
| All monocytes | 92.6 |

## ACKNOWLEDGEMENTS

The author and TheScientificWorld wish to thank Axis-Shield PoC, AS, Oslo, Norway for their kind permission to adapt OptiPrep™ Application Sheet C9 in the preparation of this Protocol Article.
